# Supplementary material for: Are carbon emissions trading and green financial instruments synergistic? -Comprehensive quantitative research based on content analysis
Source: PLoS One. 2024 Mar 7;19(3):e0298601. doi: 10.1371/journal.pone.0298601 (PMC10919601; doi:10.1371/journal.pone.0298601)
Supplement: S1 Data — (ZIP) [file pone.0298601.s001.zip › Data/地级市Z_F工作报告.zip!/║■─╧/╔█╤⌠╩╨/└┤╫╘╣1⁄2╓┌║┼í╛┬φ┐╦╩2╛▌═°í┐_2014.docx]

各位代表：

现在，我代表市人民政府，向大会作工作报告，请予审查，并请各位政协委员和列席人员提出意见。

一、2013年工作回顾

过去的一年，在省委、省政府和市委的坚强领导下，在市人大和市政协的监督支持下，我们顶住全国、全省经济下行的压力，战胜历史罕见的持续高温干旱，围绕“八个建成”，促进“三个高于”，开拓进取，扎实工作，基本完成了市十五届人大一次会议确定的目标任务。

1、经济持续较快发展。实现地区生产总值1130亿元，增长10.2%，三次产业比由上年的24.5：38.7：36.8调整为22.5：38.9：38.6。财政收入迈上百亿台阶，达到104.3亿元，增长22.6%。工业实力增强。全部工业总产值达到1742亿元，建材、食品两大产业产值过200亿元，其中建材产业216亿元、食品产业210亿元，均增长18%。规模工业增加值达到414.7亿元，增长12.4%。新增规模工业企业114家，总数达到880家。产值过20亿元企业4家、过10亿元8家、过亿元435家。5家市级园区获批省级工业集中区，实现一县一省级工业集中区。宝庆工业集中区机构正式组建，下放管理权限23项，理顺市、区、园区管理体制，连片征拆取得了突破性进展，新建标准厂房60万平方米，一批重点工业项目正在抓紧建设，实现技工贸总收入60亿元，正式启动了申报国家级经济开发区。农业生产稳步发展。面对历史罕见的严重干旱，全市组织万名干部下村抗旱，组织专业队伍和干群扑灭山林大火，确保了粮食播种面积910.9万亩、总产量343万吨，确保了人畜饮水安全，确保了大旱之年的大丰收。全年完成经济作物面积245万亩。完成烤烟收购24.9万担。隆回试验超级稻亩产988.1公斤，再次刷新世界纪录。绥宁被认定为国家级杂交水稻种子生产基地。面对全国性的禽流感和复杂的动植物疫情，我们及时采取有效措施，防控和处置了人感染H7N9禽流感及其他动植物疫情。农业产业化企业达到4116家，其中规模企业243家、新增33家，实现农业产业化销售收入310亿元，增长10%。农业综合机械化水平达到54%，比上年提高6个百分点。现代服务业日趋活跃。实现社会消费品零售总额431.9亿元，增长14%。建成全国农产品现代流通综合试点城市项目6个，标准化改造农贸市场40个。完成旅游产业总体规划修编，成功举办第二届中国大桂林旅游·湘桂原生态风情节和第八届湖南西部农博会。新宁跻身全省首批旅游特色县域经济三个重点县之一，并成功创建全国旅游标准化示范县。旅游人次达到1900万，增长16.7%，旅游总收入140.2亿元，增长17.8%。本地银行实际新增贷款138.9亿元，增长38%，外地银行新增贷款22.2亿元，增长212.5%。本地银行新增存贷比首次突破60%、达到62.5%，余额存贷比42.7%、比上年提高2.9个百分点。新开业小额贷款公司5家。市本级新增金融机构5家。直接融资10亿元。实现保费收入36.5亿元，增长10.8%。

2、项目建设掀起热潮。固定资产投资首次突破千亿，达1030亿元，增长35.2%。实施重点项目274个，新增项目为历年之最，达84个，完成投资415.8亿元，增长63%，其中“四个十大建设工程”完成投资187亿元。九兴鞋业一期600万双耐克鞋生产线、三一湖汽一期工程、湘窖酒业2万吨清香型白酒生产车间、李文集团九盛食品等一批重点工业项目竣工投产。完成132座重点小二型病险水库除险加固，整治91座一般小二型病险水库。建成高标准农田9万亩，改造中低产田2万亩，改良草山1.1万亩。打造新农村建设示范工程试点镇12个、示范片20个、示范村200个。洞新、怀通高速公路的竣工通车，使我市境内高速公路通车里程达到418公里，居全省前列，实现县县通高速，安邵、邵坪高速抓紧建设，武靖高速即将开工，我市真正进入了高速新时代。邵永铁路扩改列入“十二五”建设规划，沪昆高铁建设和娄邵铁路扩改施工顺利，全市人民多年来努力争取的怀邵衡铁路正式获批并全线开工，标志着我市铁路建设进入高速发展期。武冈机场开工建设，开启邵阳航空新纪元。启动邵东军民合用机场规划编制。改造国省干线206公里、危桥41座，建成农村公路859公里、乡镇客运站26个、招呼站1091个。学院路、桃源路、立新路三期、邵水西路中段、五一南路延伸段、邵水东路六中段建成通车，东互通世纪大道、市行政中心、桃花路、邵石南路、雪峰大桥、大祥立交桥、西苑公园、爱莲池公园、湘林大酒店等项目进展较快，桂花大桥、蔡锷故居整体开发及周边路网工程开工。南山风电二期并网发电，新宁风雨殿、隆回宝莲风电正式投产，洞口苏宝顶风电开工。新建220千伏输变电工程2个、110千伏输变电工程4个。市体育中心、文化艺术中心、中心医院东院、邵阳报业大厦、职工维权帮扶中心开工建设。市第一人民医院儿科综合楼基本建成，市脑科医院门诊和住院综合楼投入使用，邵阳幼师专科学校完成可研批复。

3、城镇面貌明显改观。编制完成东部城镇群城镇体系规划、城市总体规划和各层次专项规划，规划引领作用不断增强。中心城市建设加快。城市土地利用总体规划修改获批，建设用地总规模新增20平方公里，市中心城区规划面积达到120平方公里，为建成“双百”城市奠定了空间基础。最美十条街完成规划设计，敏州西路、双坡路、蔡锷路等完成美化绿化，南互通敞口工程抓紧推进，资江南岸风光带片区整体开发正式启动。改造小街小巷367条，市民家门口路平灯亮的夙愿得以实现，彻底改变了晴天一身灰、雨天一脚泥的状况。灯化亮化工程初见成效，城市夜景靓丽夺目。1206人防工程竣工营运。管输天然气入邵工程竣工通气。桂花渡水厂取水改造和管网配套工程进展较快。洋溪桥污水处理厂扩建配套管网工程、江北污水处理厂一期工程开工建设。新建垃圾中转站28座，更新垃圾桶、果皮箱3000个，垃圾无害化处理率达到90%以上。城区管理更加规范。市区环境卫生、渣土运输、户外广告和园林绿化等城市管理工作进一步规范。通过宣传劝导、回购摩托车、安排就业、新增公交线路6条、学生专线13条、公交车113台、出租车320台、开工建设4个公交停保场、初步建成公共自行车服务系统等措施，平稳、有序、成功实施了中心城区“限摩限电”专项整治，极大地改善了交通状况，有效防止了“两抢一盗”，大大减少了城区交通事故，方便了广大市民的出行。“限摩限电”的成功做法，受到省领导充分肯定，省内外10多个兄弟市州来邵考察学习，中央和省多家主流媒体竞相报道。控违拆违强力推进，拆除违法建筑11.5万平方米。城镇建设同步跟进。批回新增建设用地2.5万亩，增长51%，其中市本级8156亩，增长77%。市区征地7967亩、征收集体土地上房屋26万平方米。城镇化率达到38.6%，比上年提高2.5个百分点。建筑业和房地产业稳步发展，全市商品房竣工面积186万平方米、增长10%，销售面积220.6万平方米、增长30.5%。9个县(市)城基础设施不断完善，综合承载能力进一步提升。小城镇建设各具特色，17个镇被列为全省中心镇和特色镇，洞口县高沙镇被授予全省两型示范镇。

4、改革开放强力推进。36户市属国有工交企业有27户进入改革程序，25户完成职工安置费发放，安置职工2.1万人。成功组建市国有资产投资经营公司，筹集资金10.9亿元。5个有改制任务的县市启动22户国有企业改革，完成14户。国有流通企业和集体企业的改制抓紧进行。大圳灌区改革改制基本完成。征地拆迁安置改革进展较好，市区全面实施集中安置模式。开工建设11个安置房小区，解决7000多户失地农民的住房问题。正式出台失地农民保障措施，彻底解决失地农民的生存和发展问题。基本完成大圳灌区改革改制，医药卫生、财税金融、水利管理、殡葬秩序、资源环境价格和农村综合改革等也都有序推进。引进内资589.6亿元、外资1.37亿美元，分别增长20.5%、20.4%。进出口总额5.9亿美元，增长25%。新批境外投资企业25家，建成对外劳务输出平台2个，拥有国际工程承包资格企业4家。韶山海关邵阳监管组、株洲出入境检验检疫局邵阳工作组入驻办公，开通了一般报关报检业务。

5、民生民利有效改善。城镇居民人均可支配收入17647元，增长10%，农民人均纯收入6933元，增长12.5%。新增城镇就业5.1万人、农村劳动力转移就业6.2万人，零就业家庭动态援助达到100%。企业退休职工人均每月增加养老金170元。新型农村养老保险参保374万人，发放养老金6.5亿元。保障农村五保户6.3万人，发放供养金1.7亿元。改扩建乡镇敬老院23所。完成农村危房改造和民房恢复重建14469户。新建廉租房4459套22.1万平方米、公租房12526套67万平方米，实施城市棚户区改造8128户44.1万平方米，改造林场危旧房8666户。归集住房公积金11.5亿元、发放贷款10.3亿元，分别增长32%、30%。邵阳市武陵山片区发展与扶贫攻坚规划正式获批，1734个项目列入规划，已落实教育、交通、扶贫等优惠政策近20项，我市与国开行签署金融支持产业扶贫合作协议，第一期为我市提供5亿元贷款支持。整合各类扶贫资金6.7亿元，减少13万贫困人口，使我市贫困人口从前年的190万人减少到去年的177万人。完成饮水工程97处，解决40万农村人口饮水不安全问题。5个小型农田水利设施重点县建设项目通过验收。物价监管调控有力，居民消费价格涨幅控制在3%以内，我市被评为全国首届价格工作先进单位。28项为民办实事指标圆满完成，其中16项超过省定目标。

6、社会事业全面进步。科技创新力度加大。新增高新技术企业6家，实现高新技术产品增加值120亿元。转化科技创新成果18项。申请专利1200件，授权798件。新增中国驰名商标6件、国家地理标志证明商标3件。建成院士专家工作站2家。邵东被评为全国知识产权示范县，邵东、新邵荣获全国科技进步先进县。各类教育趋于协调。基础教育得到巩固，职业教育规模扩大，民办教育蓬勃发展，高等教育办学水平提高。建设了20所农村公办幼儿园和136所省级义务教育合格学校。新建农村教师周转房660套。文体事业健康发展。文化产业逐步壮大，实现增加值47.8亿元，占GDP的4.2%。“送戏下乡”演出573场，放映农村公益电影6.2万场次，5.5万户农村居民通了广播电视。建成农家书屋5542个，实现每个乡镇均有文化站。“三馆一站”免费开放。祁剧《梦蝶》入选国家舞台艺术精品工程。新增全国重点文物保护单位6处，9个村入选中国传统村落名录。绥宁侗寨申遗的侗寨保护规划、遗产研究及环境整治取得阶段性成效。文化旅游产业园列入全省文化产业十大重点项目。加大文化市场整顿力度，坚决打击无证照娱乐场所噪音扰民行为，文化市场进一步规范。竞技体育取得好成绩，邵阳健儿在省以上竞赛中获金牌24块，其中国家级金牌2块。成功举办全市第三届少数民族传统体育运动会。人口卫生服务水平提高。市中医院成功创建三级甲等中医医院，市中西结合医院、新邵正大骨科医院通过三级甲等医院国家评审。11类基本公共服务项目和5类重大公共卫生项目全面落实。7个县级医院、36个乡镇卫生院、76个村卫生室改扩建项目竣工。197家乡镇卫生院、5133家村卫生室实施基本药物制度，实施率分别达100%、96.6%。17个乡镇卫生院和4个社区卫生服务中心全面启动乡村医生签约服务工作，25%的行政村开展乡村医生签约服务。新农合参保614.5万人，参合率97.3%。武冈市成功创建国家级卫生应急综合示范县市。人口自然增长率控制在7.5‰以内，企业法人计生创新工作经验在全省推广，人口计生工作连续四年被评为全省先进。社会治理创新成效明显。坚持用群众工作统揽信访工作，建立乡级群众工作办公室225个、村级群众工作站4231个，化解了一批突出信访问题，群众工作经验在全省推介，“洞口经验”得到了党中央和省委的充分肯定。基层民主政治建设扎实推进，群众性自治组织实现全覆盖。深入开展安全生产大检查百日行动和“打非治违”专项行动，安全生产事故起数、死亡人数和主要相对指标均实现下降。“六五”普法扎实推进，市民法制学校建设成效明显。扎实推进平安建设，依法打击“两抢一盗”等各类违法犯罪，破获一批有影响的刑事案件，公众安全感明显增强。

7、生态环境继续好转。造林36.3万亩，森林覆盖率58.5%，比上年提高0.9个百分点，其中“三边三区三年”绿色行动植树3.1万亩，是近10年造林总和。积极创建省级园林城市，市区绿地率、绿化覆盖率分别达到31.5%、36%，人均公共绿地面积8平方米。新增湿地保护面积9872公顷。完成中小河流治理项目18个，治理河道230公里，治理水土流失22.3平方公里，生态修复36平方公里。河道采砂权整体拍卖出让，综合整治成效明显。投入资金4000万元，用于矿山地质环境恢复和治理。新建户用沼气池8413口。隆回被评为全国国土资源节约集约模范县。关闭50家污染问题突出企业，淘汰73条落后生产线，完成市区龙须塘工业片区环境综合整治规划和启动工作，全年主要污染物排放总量控制在省政府下达的目标之内，万元生产总值能耗下降3%。北塔、洞口、邵阳县农村环境连片综合整治项目顺利通过验收。新宁、洞口分别被评为全国绿化模范县和全国绿色生态蜜橘示范县，绥宁纳入国家重点生态功能区范围，隆回县虎形山瑶族乡创建国家级生态乡镇，绥宁花园阁、城步白云湖、隆回魏源湖、邵阳县双江口获批国家湿地公园。

同时，统计、工商、质检、移民、老龄、市志、档案、贸促、盐业、电力、铁路、石油、烟草、人事编制、民族宗教、外事侨务、食品药品、邮政通讯、气象水文、防震减灾、无线电管理和山林纠纷调处等各项事业和工作都取得了新的进步。国防动员、民兵预备役和双拥工作成绩显著，兵役登记工作做法全省推广，国家安全和人民防空事业不断加强，驻邵解放军和武警消防部队在抢险救灾、维护稳定和支持地方建设等方面发挥了重要作用。

各位代表，一年来，我们坚决落实市委决策部署，主动接受市人大及其常委会的法律监督，自觉接受市政协的民主监督，重大事项主动与市政协进行民主协商，加强与工会、妇联、残联、文联、科协、共青团、工商联、社科联、贸促会、红十字会等群众团体联系。办理人大代表建议234件、议案2件、政协提案534件，办复率100%。修订完善政府工作规则，建立和完善了重大行政决策机制，全面实施规范性文件登记制度，认真办理行政复议和信访“两复”案件。政府信息公开内容和范围不断扩大。廉政建设进一步加强，行政监察和审计监督力度加大，公务员队伍建设和管理日益规范。

各位代表，一年来，我市呈现经济快速发展、社会和谐稳定的大好局面，得益于省委、省政府和市委的正确领导，得益于人大代表、政协委员的大力支持，得益于全市各族人民的共同努力。在此，我代表市人民政府，向全市810万各族人民，向各位代表、各位委员，向各位离退休老同志，向关心支持邵阳发展的驻邵解放军、武警消防官兵及各界人士，向积极参与邵阳建设的投资者，表示衷心的感谢和崇高的敬意！

在总结成绩的同时，我们也清醒认识到，经济社会发展中还存在许多困难和问题：经济发展总量不大，快速发展的外部条件不充分，内在动力不足；三次产业结构不优，财政收入占GDP比重偏低，税收占财政收入比重偏低；县域经济不强；城镇基础设施欠账较多；资金、土地、资源等要素制约仍然突出；一些涉及群众切身利益的问题尚未得到根本解决；政府职能转变不够理想，发展环境有待进一步优化，等等。对此，我们一定高度重视，认真加以解决。

二、2014年工作安排

各位代表，2014年是实现“十二五”规划目标的关键之年，也是十八届三中全会后的“改革元年”。世界经济延续缓慢复苏态势，新的增长动力源尚不明朗。全国经济运行存在下行压力，但仍将保持国内生产总值合理增长。省委促进“三量齐升”，分类指导推进全面建成小康社会步伐加快。随着武陵山片区扶贫攻坚、老工业基地改造等一批重大政策深入实施，武冈机场、沪昆高铁、怀邵衡铁路等一批重点工程加快建设，我市在全国东部沿海与中西部、长江开放经济带和沿海开放经济带、湖南东部与中西部的过渡带和结合部的区位优势已经凸显，湘中西南城市的中心位置已经凸显，后发赶超跨越发展的空间优势已经凸显，全市人民想发展谋发展的精气神已经凸显。只要我们切实增强机遇意识、忧患意识、责任意识，坚持底线思维，强化问题导向，坚定信心，发挥优势，迎难而上，我市的经济社会发展将在新的更高起点上更好更快发展。

做好今年政府工作，必须全面贯彻落实党的十八大和十八届二中、三中全会以及中央、省委经济工作会议精神，继续高举加快发展旗帜，以全面深化改革总揽发展全局，紧扣“八个建成”战略目标，继续坚持稳中快进工作总基调，继续坚持“三个高于”工作总方针，继续坚持以项目建设为工作总抓手，更加注重发展实体经济，更加注重提高发展质量，更加注重提高生态文明水平，更加注重保障和改善民生，实现经济持续快速发展、社会大局和谐稳定。

今年我市经济社会发展预期目标是：地区生产总值增长11%，规模工业增加值增长14%，固定资产投资增长30%，社会消费品零售总额增长15%，财政收入增长16%，利用外资增长20%，利用内资增长25%，进出口总额增长20%，城镇居民人均可支配收入增长12%，农村居民人均纯收入增长12%，城镇化率达到40%，居民消费价格涨幅控制在4%以内，人口自然增长率控制在8.5‰以内，万元生产总值能耗下降3%，主要污染物排放总量控制在省政府下达的目标之内。

围绕上述目标和任务，政府将始终把项目建设放在经济发展第一要务的首要位置，大抓项目，抓大项目。在抓项目、抓投资、抓招商引资上，继续解放思想，继续营造投资优势和洼地，继续优化项目建设环境，以思想换项目，以政策换项目，以优势资源换项目，以优良资产换项目，克服懒政和惰政思想，用抓铁有痕、踏石留印的狠劲和吃得苦、耐得烦、霸得蛮的宝牯佬精神，全面推进基础设施项目、产业项目、民生项目等的投资建设。今年全市实施重点项目305个，总投资2200亿元，比去年新增项目31个。其中产业项目投资占投资总量的比重不低于40%。

今年突出抓好以下十方面工作：

1、始终把壮大工业作为经济发展的重中之重

我市经济发展的短板在工业，潜力也在工业。壮大工业应成为我们长期坚持、不懈努力的重中之重工作。今年工业项目投资占产业投资比重达到60%。实施开工、投产、招商、新增均过百家的“四百”计划，实现年产值过50亿元企业1家、过10亿元15家、过5亿元40家。

实施园区经济倍增计划，开展“工业园区建设年”活动，出台支持园区发展的政策，编制完成各园区建设总体规划，理顺管理体制，搭好融资平台，完善基础设施，强力推动园区集聚集约发展。园区规模工业增加值增长18%以上，占全市规模工业增加值比重达到60%。市本级重点整合宝庆工业集中区、邵阳经开区和龙须塘化工区，抓紧申报国家经济开发区，快速推开宝庆工业集中区内邵阳大道两旁项目建设，强力推进连片征拆工作，建成一批连片标准厂房，引导企业退城入园，加快服饰、食品、医药、发制品、中小微创业园等特色园区建设，引进一批战略投资者，争取完成固定资产投资50亿元，其中工业项目投资28亿元，技工贸总收入达到200亿元。

出台扶持规模工业企业发展办法，重点调度86家规模以上新建和续建工业项目，对重点骨干企业实行“一企一策”帮扶，协调好煤、电、油、运、气等要素供应，积极帮助企业解决融资、招工、人才和拓展市场等问题。推行品牌战略，培育和申报一批省级、国家级品牌。支持矿产、五金、皮具、箱包、液压、发制品、小水电、机械制造等产业抱团发展，组建企业集团，支持企业延长产业链发展，支持拥有自主知识产权的科研成果产业化、基地化，培育一批具有地方特色和竞争力的骨干企业。积极承接工业企业产业转移，大胆引进和支持世界500强和国内500强工业企业来我市建设生产基地，兼并重组我市中小企业，扩大市场销售份额。注重发挥中小企业投资见效快、经营机制活、吸纳就业多的优势，做好整合文章，推进转型升级，增强自我发展能力。

2、夯实农业基础，大力发展现代农业

推进农业产业化。稳定粮食产量，大力推广优质稻、双季稻，确保粮播面积910万亩、总产340万吨。实现经济作物总面积245万亩、年产值18亿元，种植烟叶10万亩以上，收购烤烟30万担。以发展标准化农业为抓手，继续推进粮油“百亿产业”工程，启动市城区粮食仓库整体搬迁，建设大型粮油及农副产品批发零售市场。推进标准化规模养殖建设。加强城镇蔬菜基地和特色蔬菜基地建设，实施好“菜篮子”工程。出台扶持农副产品深加工企业的发展政策，新增农产品基地10万亩，扶持农业产业化龙头企业和农民专业合作组织100家，组织实施产业化经营项目39个，争取新增农业产业化规模企业20家。启动农业科技产业示范园建设。做好南方金银花改版正名工作。强化农产品质量安全，创建标准化示范县1个、示范乡镇20个、示范基地36个，新增无公害农产品15个、绿色食品23个。加大渔政管理和野生动植物保护力度，做好动植物疫病防控工作。

夯实农业发展基础。启动水利建设五年行动计划，加快衡邵干旱走廊综合治理，推进崀山水库、木瓜山第二水源建设工程。完成3个中型水库、91座重点小二型水库、116座一般小二型水库除险加固任务，启动团结坝、施家坝2个大型水闸建设，新建隆回东风水库。抓好“五小”水利工程建设，逐步解决“最后一公里”问题，修复水毁工程1.4万处，加固堤防55公里。治理水土流失11.1平方公里，推进4个小型农田水利设施重点县建设。加强人畜饮水工程建设，解决37万农村人口饮水不安全问题。扎实实施娄邵盆地农田基本建设项目，以此为基础，整合水利、农业、林业、烟草等涉农资金，建设高标准旱涝保收农田33万亩，改良草山1.2万亩。出台支持农业机械制造和使用的政策，发展农业机械制造业，推广各类农机具4万台，争取农业生产综合机械化水平达58%，水稻生产全程机械化水平达63%。

抓好新农村建设。积极探索多元投入、管理、运营的新机制，加大公共财政对农村基础设施建设的覆盖力度，促进水、电、路、气、通信、污水和垃圾治理等基础设施建设。创新农业经营体系，大力培育专业大户、家庭农场、农民专业合作社等新型经营主体，完善“企业+基地+农户”农企联结机制。健全农产品质量安全检测、监管、诚信体系，确保食品安全。千方百计增加农民工资性、经营性收入，探索农民增加财产性收入渠道。推进农村危房连片改造，打造新农村建设示范片20个、示范村200个。

抓好村庄规划编制和实施，引导农民按规划建房。探索村级集体经济增收新途径，不断提高村级集体经济发展水平，力争四年内消除村级集体经济空白村。

3、着力推进新型城镇化建设推进东部城镇群和西部生态圈建设。争取东部城镇群城镇体系规划尽快获省里批复，完成高速公路围合区域城乡一体化规划。加快编制东部产业带规划。启动东部城镇群快速路环立项可研工作。改造西湖北路，拉通学院路延伸段，抓紧市区经邵东机场至邵东县城一级快线建设，启动市区至隆回快速通道前期工作。加快邵坪高速L2、L3、L4、L5的建设，确保沪昆高铁邵阳站前广场年内竣工使用。完成怀邵衡铁路沿线城市的客站、货站及其周边区域的规划和前期工作。在西部旅游发展规划的基础上，完成西部生态圈综合规划。

加快中心城市基础建设，强化中心城区的管理。投资60亿元，实施一批新建项目和111个续建项目，加快宝庆新城、桃花新城、滨江新城和北塔新城建设。以路网建设为核心，抓紧建设外环线，拉通邵水东路、邵水西路、魏源西路、邵石路、电机路、新华路等瓶颈路，打通新华南路大坡岭隧道和建设路东塔公园隧道，竣工东互通世纪大道、双坡南路、敏州西路、铁砂岭路、南互通敞口改造工程，全面开工建设最美十条街和白马大道、雪峰北路等一批道路工程，推进雪峰大桥、桂花大桥、大祥立交桥建设。加快西苑公园、六岭公园、北塔公园、资江南岸风光带片区建设。启动工人文化宫片区改造。抓好昭阳路、东邵路、紫霞路、雪峰南路、白马大道、新城大道与怀邵衡、娄邵铁路的下穿立体交叉工程配套规划与建设。完成1213人防主体工程，推进市人防疏散隐蔽基地建设。完成江北污水处理厂厂区和部分配套管网工程，推进洋溪桥污水处理厂改扩建、燃气安全指挥中心建设，开工建设污泥集中处置中心工程，支持自来水厂提质改造。发挥天然气入邵工程效益，新建燃气主干管50公里，改造60公里。对违法建设实行零容忍，强力推进控违拆违，发现一起拆除一起，实现规划圈内违法建设零增长。积极创建国家卫生城市，建成省级文明城市和园林城市。继续推进城市管理综合整治，强化渣土管理，扩大城区亮化范围，规范城区广告招牌，建成数字城管系统，逐步缓解交通拥堵和停车难问题，使城市管理水平再有一个大的提升。

支持县城和小城镇建设。推进城镇化建设，必须坚持走以人为本、四化同步、科学布局、绿色发展、文化传承的新型城镇化道路，保持市中心城市、县级市县城以及小城镇的协调发展。支持商品房开发建设，推动房地产业健康发展。启动天然气全覆盖、县县通工程。鼓励9个县(市)城按照各自规划，加快扩容提质步伐，建成区域经济、文化、政治中心。按照挖掘潜力、完善功能、扩大规模、凸显特色的要求，打造一批特色小城镇，抓好廉桥、高沙、崀山、陈家坊、金石桥、五峰铺等中心镇、示范镇开发建设，支持金石镇、下花桥镇建成山水园林式特色镇。探索县城和小城镇管理市场化新模式。

抓好城市建设和管理，必须建立和完善一系列体制机制，必须把老城改造提质与新城建设统筹考虑，必须使园区建设与新城建设融合协调，必须使新城扩容与居民和农民进城就业相适应。强化规划意识，提高规划水平，增强规划的权威性。在政府性债务可控的前提下，扩大融资规模，完善融资平台，开拓融资渠道，创新融资方式，确保项目建设资金不断链。越是用地紧张，越要珍惜土地。清理闲置土地，启动市城区火车货站搬迁和铁路连接线的闲置清理工作。扩大用地规模，确保市域内土地占用增补平衡。解开征地拆迁难题，理顺市区两级征地拆迁体制，强化区级政府主体责任，采用经费包干模式，统一征地拆迁政策，推行依法征拆、阳光征拆。落实已出台的失地农民保障政策，竣工一批拆迁农民安置房，让80%已拆迁户住上房，逐步进入先安置后拆迁的良性循环。进一步明确城市管理的责任主体，权、责、利向基层倾斜，大胆推行政府购买管理和服务，提升城市管理水平。

4、大力发展现代服务业

壮大旅游产业。召开全市旅游发展大会，出台加快旅游发展的意见，积极引进战略投资者开发旅游，着力培育一大批旅游“六要素”市场主体。支持崀山建成5A景区，南山、云山、花瑶、那溪、白水洞、蔡锷故居、紫薇博览园创建4A景区，争取黄桑成功申报为国家生态旅游示范区。加快新宁旅游特色县建设。重点开发宝庆竹刻、蓝印花布、滩头年画、花瑶挑花、武冈卤菜、墨晶石雕等具有邵阳特色的旅游商品。把以招揽游客为目的的宣传促销放在更加突出位置，多形式、全方位开展旅游促销活动，承办好湖南国际旅游节和大湘西旅游经济协作区合作联盟年会，提升邵阳旅游的知名度和美誉度。全年接待游客2100万人次，实现旅游总收入160亿元以上。

加快商贸物流建设。围绕打造湘桂黔边陲物流中心，着力推进湘西南国际商贸物流城、怀邵衡铁路货站及物流体系、大型商业购物中心、国家农副产品流通示范城市、汽车贸易城、建材城、廉桥药都和湘西南医药物流园、烟草物流园等重点项目建设，争取锑交易中心落户新邵。推进“万村千乡市场工程”建设，建成乡镇商贸中心3家、直营店50家以上，标准化改造农贸市场30家。继续培育限额以上商贸流通企业，加快培育一批大型物流、居民服务、再生资源回收、烟草物流、药品流通等市场主体，依法严厉打击制售假冒伪劣产品、价格欺诈等不法行为，确保社会消费市场健康有序。

大力发展新兴服务业。支持发展电子商务、信息服务、现代物流等生产性服务业，推动总部经济、金融服务、会展经济、软件和服务外包等产业发展。推动科技研发、创意设计、信息咨询等新兴产业发展。

5、以交通和能源建设为重点，夯实快速发展基础

继续完善立体交通网络。加快安邵、武靖、邵坪高速及其连接线建设进度，力争开工建设白新高速，做好安北高速邵阳段开工前准备工作，积极推进崀山至资源、洞口至溆浦高速的建设，开展城步至龙胜高速前期工作。加快怀邵衡铁路建设，基本完成娄邵铁路扩改和沪昆高铁邵阳段建设。启动邵永铁路扩改工程前期工作，以及靖永郴、张常桂、冷邵、娄底至邵阳城际铁路等项目前期规划研究工作。加快武冈机场场内外工程建设，启动邵东军民合用机场预可研工作。建成邵塘一级公路、长河二级公路，加快建设六金、八老、新烂、罗五、陶黄及衡邵高速新邵连接线6条干线公路、206公里，新开工隆武、金狮、边南等6条干线公路、107公里，完成9个干线公路项目207公里前期工作。继续抓好农村公路建设，完成渡改桥3座、危桥改造33座。开展邵阳港前期工作。加快建设武冈汽车北站等4个汽车站，开工建设新邵坪上、市区火车南站两个大型客运枢纽。做好农村客运站场建设扫尾和农村客运招呼站建设工作。启动市城区汽车东站和南站搬迁工作。

提高能源保障水平。争取宝庆煤电二期工程立项，开工建设五团、龙山、望云山、宝莲二期等9个风电场项目，推进垃圾发电、光伏发电、生物质能发电项目。新扩建110千伏输变电工程10个、35千伏输变电工程14个。加快市区至各县市的天然气管网和汽车加气站建设，配套城镇燃气基础设施，不断提高天然气普及率。保障石油安全供应。

6、深化改革开放，激发发展活力

坚决完成国企改革任务。在去年工作基础上，继续按照统一政策、多渠道筹措资金、全方位关心下岗职工等工作要求，及时足额支付改制企业职工安置等各项费用，确保全面顺利完成剩余11户市属国有工交企业改革任务。认真落实省委、省政府在我市率先关闭退出小煤矿的政策精神，坚决平稳关闭小煤窑60对。因企制宜，基本完成其他市属企业改革。督促指导武冈、绥宁、邵东、洞口和邵阳县尽快按计划完成所属国企改革任务，圆满完成国企改制三年攻坚计划。与此同时，强化对国有和国有控股企业的绩效考核和财务监管，积极探索集中统一监管经营性国有资产的办法。

深化财税金融改革。改进预算管理制度，实行专项资金预算绩效管理。全面推进政府部门“三公”经费公开。深入推行公务卡结算改革，规范公务消费行为。深化政府采购制度改革，加快政府采购管理交易平台建设，推进电子化政府采购。制订出台税收保障办法，稳步实施“营改增”，强化税收征管。大力引进股份制银行来邵设立分支机构。加快组建邵阳农商银行，启动农村商业银行筹建工作。争取直接融资20亿元以上、间接融资180亿元以上，本地银行新增存贷比继续保持在60%以上。推进投融资体制改革，做大做强城建投，搭建好交通投、教育投、水利投、国资投等融资平台。促进保险市场健康发展，保费收入增长10%以上。

扎实推进农村综合改革。加大土地流转改革力度，探索土地流转交易市场建设，完成农村集体土地所有权登记发证，鼓励承包经营权向专业大户、家庭农场、农民合作社、农业企业流转。继续完善集体林权、产权制度改革，强力推进农村房屋产权、林权和土地使用权等不动产统一登记。

加大招商引资力度。围绕优质资源、优良资产和优势产业，精心开发包装招商项目，创新招商方式，加快塑造包括劳动力、土地、发展环境、产业配套等在内的新的比较优势和竞争优势，吸引更多客商来邵投资兴业。充分利用国家商务部在城步定点扶贫的契机，借助其资源，推动承接产业转移和开放型经济发展。做好引老乡回故乡建家乡工作，办好2014年邵商大会，做好2015年湘商大会筹备工作。

加快外贸发展平台建设。以龙头企业为支撑，加快鞋业、打火机、发制品、箱包服装等十大出口基地建设。确保发制品产业园建成投产，争取打火机产业获批“国家外贸转型升级专业型示范出口基地”。启动邵阳海关、出入境检验检疫局大楼主体工程建设，加快公路口岸建设，打造大通关平台。

7、做大做强县域经济

以分类指导各县市区加快推进建成全面小康为总抓手，出台加快县域经济发展的意见，结合各县市资源禀赋、区位条件、产业结构和发展水平，实行差异化政策，大力发展特色经济，涌现更多特色县域经济重点县。以统筹城乡一体化发展为目标，以改革开放为动力，整合各类资源、资金和力量，着力推进县域产业发展、基础设施建设、城镇化建设、扶贫攻坚和生态文明建设，促进县域经济你追我赶、竞相发展。推行简政放权，进一步理顺市县两级体制，扩大县级自主权，使事权、财权与自主决策权相匹配。积极鼓励各县市区对照省里考核指标，认真抓好工作落实，在全省全面小康、县域经济考核中位次前移。力争新增财政收入过10亿元、GDP过100亿元的县市3个。

全面推进武陵山片区区域发展与扶贫攻坚实施规划，着重抓好列入规划的项目建设，落实与国开行合作的金融支持措施。坚持精准扶贫，实行一乡一策、一村一策、一户一个脱贫计划。强化产业扶贫，统筹保障贫困地区基本公共服务，抓好贫困地区教育工作，不让贫困地区的孩子输在起跑线上，推动水、电、路、气、房、环境整治“六到户”。全年减少15万贫困人口。

8、统筹推进社会事业发展，切实保障和改善民生

促进社会事业全面进步。鼓励企业积极参与科技研发，加大科研投入，引进科技人才。新增高新技术企业6家以上，高新技术产业产值突破600亿元，实现专利申请1300件，授权850件，建成院士专家工作站4家。抓好科技成果推广、转化和运用。推进教育强市行动计划，促进教育均衡发展。以建好168所合格学校为重点，全面改善义务教育学校特别是农村边远薄弱学校办学条件。合理配置城区教育资源，启动城区化解大班额三年行动计划，逐步解决城区大班额问题。抓好校车安全管理。组建职教集团，建设“职教新城”。完善特殊教育体系，支持市特殊学校搬迁工作。加快邵阳电大、市一中异地搬迁步伐。扎实推进邵阳幼师专科学校组建工作，不动摇做好邵阳学院和邵阳医专合并工作。加强“三通两平台”教育信息化建设。全面实施新课改，着力提高教育质量。进一步强化师德师风建设。继续开展送戏下乡、农家书出版物补充更新、农村公益电影放映、广播电视村村通四大文化惠民工程，加大文化遗产保护力度，继续做好绥宁侗寨申遗工作，推进非物质文化遗产生产保护基地等重点项目建设，加快市文化艺术中心和文化旅游产业园建设，进一步壮大文化产业，提高文化产业产值占GDP比重。做好《邵阳文库》编撰工作。推进市体育中心建设，努力申办全省第十三届运动会，积极组团参加全省第八届少数民族传统体育运动会。深化医药体制改革，抓好医疗保障、医疗服务、公共卫生、药品供应保障建设，加快市中心医院东院建设并建设市传染病院，推进二人民医院异地扩建工程，开工市中医院肿瘤综合楼、全科医师培训基地。坚持计划生育基本国策，推进基层计生体制创新，稳步实施“单独二孩”生育政策，依法管理生育行为，促进人口长期均衡发展、计生家庭和谐发展。

扩大就业和完善社保体系。加快创业孵化基地和就业见习基地建设，出台就业、鼓励创业和引进人才的政策措施，吸引更多的高校毕业生特别是本籍大学生、研究生回乡就业和创业，坚决一个不漏地解决纯农户大学毕业生的就业问题，努力创建省级创业型城市。实施下岗职工再就业“清零”计划，创造更多就业岗位解决零就业家庭的就业问题。新增城镇就业4.9万人，失业人员再就业2.1万人，完成各类就业培训5.9万人，转移农村富余劳动力稳定在130万人以上。健全社会保障体系，推进社保扩面工作，全面落实“老工伤”待遇，提升社保基金统筹层次。按政策提高城乡低保标准，实现应保尽保。抓好敬老院、老年公寓、残疾人康复中心建设。围绕“两房两棚”、“两供两治”等人民群众关心的实际问题，集中力量为民办实事。实施城市棚户区和工矿棚户区改造五年行动计划，逐步解决居民职工住房困难问题，年内完成廉租房4190套21万平方米、公租房13400套80万平方米，改造城市棚户区20200户109万平方米、国有工矿棚户区960户4.8万平方米。争取归集住房公积金13亿元，发放个人住房贷款11亿元。

切实维护社会和谐稳定。推进社会治理创新，逐步完善社会治理格局。进一步加强和改进政法机关基层基础工作。坚持“治邵必严”方针，完善社会稳定风险评估机制，深入开展“打黑除恶”专项行动和治安重点整治，严厉打击非法集资等违法犯罪行为，加强社区管控，深化禁毒工作，提升群众安全感和满意度。抓好“六五”普法工作和市民(村民)法制学校建设，增强全民法制意识。重视网络正面宣传，强化网络监管。进一步完善群众工作体制机制，充分发挥群众工作站作用，抓好矛盾纠纷排查化解，全面提升信访工作和群众工作水平。落实党政领导干部安全生产“一岗双责”，落实企业主体责任，加强县乡安全生产监管能力建设，深化安全隐患排查整治，扎实开展“打非治违”行动，确保安全生产事故指标不超省控要求，争创全省安全生产先进单位。抓好防灾减灾体系建设，提高气象、地质、地震灾害防御能力。加强市场价格、产品质量、粮食流通和食品药品安全监管，维护正常市场秩序和群众身体健康。

9、以建成湘西南生态屏障为目标，努力打造水更清、山更绿、气更净的生态环境

实施绿化美化工程。着力提高森林覆盖率，深入推进“三边三区三年”绿色行动，争取造林36万亩，绿化公路300公里、河道150公里。加强城市绿化规划编制，严格实施绿线管制制度，开展城市绿荫行动，提高公共绿化管护水平，创建国家森林城市，争取我市纳入全国生态文明建设先行示范区。巩固西部县市生态优势，抓好楠竹低改和特色资源培育。支持北塔打造两型社会示范区。开展生态县、生态乡镇、生态示范村创建活动。继续实施城乡清洁行动，加强农村环境综合整治，逐步改善村容环境卫生。

大力开展节能减排。提倡低碳生活，争取全社会综合能耗下降3.5%、新建建筑节能设计率和审查合格率100%。认真实施“十大环保工程”建设，重点抓好龙须塘、洋溪化工区综合治理，全力推进污染物总量减排工程，不再批准达不到排放标准和总量控制目标的项目，关闭一批高污染、高耗能企业。抓好新宁低碳经济试点工作，加快新邵雀塘循环经济产业园建设，发挥隆回循环经济示范效应。开展节水型城市创建工作，加强饮用水水源地保护，抓好资江、邵水流域清淤和污染防治，完成13个中小河流治理项目。推广清洁能源，新建户用沼气池5000户、大型沼气工程3处。

与此同时，认真抓好第三次全国经济普查和第一次全国可移动文物普查。加强4G网络建设。推进补建的空白乡镇邮政所开通运营。深入开展“双拥”活动，强化国家安全观念，支持人民解放军和武警消防部队建设，抓好国防动员和民兵预备役工作，进一步落实军人优待抚恤政策，做好军队转业干部和复员退伍军人接收安置工作。

10、以践行党的群众路线教育实践活动为契机，加强政府自身建设。按照“照镜子、正衣冠、洗洗澡、治治病”总要求，针对“四风”问题，深入开展以为民务实清廉为主要内容的党的群众路线教育实践活动，着力解决人民群众反映强烈的突出问题，努力打造人民满意政府。

我们将坚持依法行政，进一步规范政府行为。推进民主法治建设，始终坚持市委领导，认真落实市委决策。自觉接受人大法律监督和政协民主监督，坚持重大事项向人大报告和政协协商制度，办好人大代表议案建议和政协提案。支持民主党派、工商联和无党派人士参政议政。充分发挥工会、共青团、妇联等人民团体的积极作用。坚持重大行政决策公众参与、专家论证、风险评估、合法性审查和集体讨论，切实提高政府科学决策、民主决策的能力。进一步规范执法自由裁量权，减少权力运作的随意性，确保规范公正文明执法。

我们将转变政府职能，进一步发挥市场决定性作用。进一步深化行政审批制度改革，简政放权，清理和减少各类领导小组等非常设机构，推进生产要素市场化，减少政府对资源的直接配置，减少对企业的干预，充分激发市场主体的创造活力。加强市场监管，更加注重权利公平、机会公平、规则公平，大力打击制假售假、商业欺诈、侵犯专利权等违法行为。解除对民营投资的约束，卸掉“弹簧门”，打碎“玻璃门”，充分激发民营资本的投资潜力。重视做好基层基础工作，充分尊重基层和群众的首创精神，加大对乡镇、村和街道、社区的经费保障力度。提高政府公共服务供给能力，保住基本、补上短板、兜好底线，促进公共服务均等化。

我们将提升政府效能，进一步优化发展环境。提高各级政府及其工作人员抓落实的能力，敢作为，勇担当，凡是政府的承诺个个兑现，凡是政府分内之事不推卸。对政府各项工作，实行目标倒逼进度、时间倒逼程序、督查倒逼落实、社会倒逼部门。出台重点项目招商、建设、管理、审计和监督的管理办法，加强行政监察和审计监督，对重点项目、重大资金实行全过程监管，堵塞项目建设中的各种漏洞，坚决惩处中饱私囊者，确保政府性投资安全规范有效。建立优化发展环境监督和责任追究机制，坚决查处不依法行政、索拿卡要、乱收费乱罚款乱摊派等不作为、乱作为、中梗阻的行为。推进社会环境整治，坚决打击一切为一己私利而设置障碍、刁难企业、非法阻工、强买强卖等违法行为，为一切投资者和经营者营造优良的创业环境。

我们将加强廉政建设，进一步促进政府清廉。全面落实党风廉政建设责任制，加强惩治和预防腐败体系建设，形成不敢腐、不能腐、不易腐的机制。推行政府及部门权力清单制度，努力把权力关进制度的笼子，着力解决发生在群众身边的腐败问题。严格规范公务接待行为，坚持厉行节约，反对铺张浪费，扎实推进公务用车改革，坚决做到政府性的楼堂馆所一律不新建、财政供养人员只减不增、“三公”经费只减不增。

各位代表，新形势、新任务对我们提出了新的更高要求，全面深化改革的新使命历史地落到了我们肩上，“八个建成”、“三个高于”的新任务现实地交到了我们手中。让我们在省委、省政府和市委的坚强领导下，真抓实干，锐意进取，为谱写中国梦的2014年邵阳篇章而努力奋斗！
